# Prevalence and associated factors of osteoarthritis and osteoporosis among Chinese adults aged 60 years or older: a multicenter cross-sectional study

**DOI:** 10.3389/fpubh.2026.1905264

**Published:** 2026-07-30

**Authors:** Qianhao Li, Guihua Miao, Zhouyuan Yang, Jing Li, Chunyan Lu, Zhirui Li, Chao Kong, Haifeng Li, Junwei Li, Shuo Sun, Qin Wang, Shuwei Ye, Xingxiao Pu, Lijun Cai, Yajie Chen, Jiarui Tang, Jiying Chen, Pengde Kang

**Affiliations:** 1 Department of Orthopedics, Orthopedic Research Institute, West China Hospital, Sichuan University, Chengdu, China; 2 Department of Orthopedic Surgery, West China Hospital, Sichuan University/West China School of Nursing, Sichuan University, Chengdu, China; 3 Research Center of Clinical Epidemiology and Evidence-Based Medicine, West China Hospital, Sichuan University, Chengdu, China; 4 Department of Endocrinology and Metabolism, West China Hospital, Sichuan University, Chengdu, China; 5 Department of Orthopaedics, Hainan Hospital of Chinese PLA General Hospital, Sanya, Hainan, China; 6 Department of Orthopedics, Xuanwu Hospital, Capital Medical University, Beijing, China; 7 National Clinical Research Center for Geriatric Diseases, Beijing, China; 8 Department of Orthopedics, Wuxi Ninth People's Hospital Affiliated to Soochow University, Wuxi, China; 9 Department of Orthopedics, Ganzhou District People's Hospital, Zhangye, Gansu, China; 10 Department of Orthopaedics, Chinese PLA General Hospital, 301 Orthopaedic Hospital, Beijing, China

**Keywords:** China, older adults, osteoarthritis, osteoporosis, prevalence

## Abstract

**Background:**

Osteoarthritis (OA) and osteoporosis (OP) are common age-related skeletal disorders in older adults, resulting in a significant burden for healthcare. However, research on the epidemiological data of bone health in older Chinese adults is still scarce. This study aimed to investigate the prevalence of OA and OP and their associated factors among Chinese adults aged 60 years or older.

**Methods:**

We conducted a multicenter community-based cross-sectional study in five Chinese cities between March 2021 and March 2023. Individuals aged 60 years or older were enrolled, and standardized questionnaires, radiographic examinations, and dual-energy X-ray absorptiometry were performed. The primary outcomes were the prevalence of OA and OP, and associated factors were evaluated using multivariable logistic regression models.

**Results:**

A total of 4,331 participants with complete epidemiologic data were included in the final analysis. The prevalence of OA and OP was 55.7% (95% CI, 54.2–57.1%) and 42.6% (95% CI, 41.1–44.1%), respectively. Female sex and increasing age were associated with higher odds of OA, while female sex, increasing age, height loss, and history of traumatic spinal fracture were associated with higher odds of OP. Vitamin D supplementation was associated with lower odds of both OA and OP.

**Conclusion:**

The findings suggested a high burden of age-related skeletal disorders among older adults in this multicenter population, highlighting the need for early screening and prevention strategies.

## 1 Introduction

With the rapid progression of global aging, the number of people aged 60 years and older is expected to reach 2 billion by 2050 (1). The latest China Census showed that 18.7% of the population is aged 60 and above (2). Although lifespan has increased with the social development, healthspan (disease-free lifespan) has not improved to the same extent. Aging is closely associated with the susceptibility to many degenerative diseases, including skeletal disorders (3, 4). According to the data of World Health Organization (WHO), falls and osteoarthritis (OA) are among the leading causes of disability in older adults. Osteoporosis (OP), a major contributor to fragility fractures, is projected to impose a substantial burden in China, with osteoporosis-related fractures expected to increase to 5.99 million and associated costs to $25.43 billion by 2050 (5). It is also reported that years lived with disability (YLD) due to knee OA in China is 968 per 100,000 individuals (6). The health and economic burden of OA and OP is increasing in China, indicating the urgent need for effective prevention and treatment strategies.

Several previous studies have reported the prevalence of OA and OP in Chinese populations. A nationwide survey by Tang et al. (7) showed that 8.1% of individuals over 45 years had symptomatic knee OA, with a higher prevalence in women (10.3%). Wang et al. (8) reported OP prevalence of 5.0% in men and 20.6% in women aged over 40 years, while Zeng et al. (9) reported OP prevalence of 6.5% in men and 29.1% in women aged over 50 years. Cheng et al. (10) using quantitative computed tomography (QCT), estimated the OP prevalence among individuals aged over 50 years to be 13.5% in men and 29.0% in women. However, most previous studies investigated OA and OP separately rather than evaluating both conditions within the same cohort. In addition, these studies differed in terms of age range, diagnostic criteria, anatomical sites, and assessment methods. For OA, previous studies mainly focused on symptomatic knee OA or single-joint involvement, whereas radiographic OA across multiple commonly affected sites has been less evaluated in older adults. For OP, previous studies have included broader adult populations, such as individuals aged over 40 or 50 years, and used different assessment methods, including DXA- or QCT-based definitions. Therefore, multicenter community-based evidence simultaneously evaluating OA, OP, and their coexistence among adults aged 60 years or older remains limited.

We conducted the Geriatric Skeleton Degenerative Disease Study (GSDDS) with support from the Ministry of Science and Technology of China. We established a multicenter community-based epidemiological database of adults aged 60 years and older from five cities representing different geographic regions of China. This study aimed to evaluate the prevalence of OA and OP and to investigate their associated factors in this multicenter older population, thereby better characterizing the burden of skeletal degenerative diseases.

## 2 Methods

### 2.1 Study design

The data for this study were obtained from the GSDDS, a multicenter community-based cross-sectional study conducted in China between March 2021 and March 2023. The study design was approved by the ethics committee of our institution and adhered to the STROBE reporting guideline. Informed consent was obtained from all participants.

Detailed methodological descriptions have been previously reported (11). In brief, participants were recruited from five cities located in different geographic regions of China (Beijing, Chengdu, Wuxi, Zhangye, and Sanya), based on the distributions of north, southwest, east, northwest, south of China. The sampling process consisted of three stages: selecting districts or counties, then streets or townships, and finally neighborhood or village committees. Eligible participants were individuals aged 60 years and older who had resided locally for at least 5 years and voluntarily participated in the study. Exclusion criteria included mental illness, mobility impairments, and communication disorders. The epidemiological questionnaire comprised five sections: baseline information, personal history, family history, history of present illness, and physical examination.

### 2.2 Data collection

Epidemiological data included sex (male, female), age (60–69, 70–79, ≥80), geographic regions (north, southwest, northwest, east, south), occupation (manual, mental), education (primary, secondary, senior), height loss (difference between self-reported peak height and currently measured height) and body mass index (BMI; <18.5, 18.5–23.9, ≥24). Additional variables included smoking and drinking habits, use of medications (glucocorticoids, anticoagulants, osteoporosis drugs, calcium supplements, glucosamine, vitamin D, activated vitamin D), sedentary time (<6 h, 6–8 h, >8 h), exercise type (single, multiple), and exercise frequency. In addition, medical history variables included diabetes mellitus (DM), hypertension (HTN), hyperlipidemia (HL), chronic heart failure (CHF), coronary heart disease (CHD), chronic obstructive pulmonary disease (COPD), chronic kidney disease (CKD), cerebral infarction, inflammatory bowel disease (IBD), peptic ulcer, rheumatoid arthritis (RA), fall-related fractures, and trauma-related fractures.

Participants underwent radiographic assessments, including X-rays of the pelvis, knee, hand, thoracic spine, and lumbar spine. Collo-diaphyseal angle (CDA) and center-edge angle (CEA) were measured in pelvis anteroposterior projection (Supplementary Figure S1). Femoral tibial angle (FTA) was measured in knee anteroposterior projection and Insall-Salvati index was measured in the lateral position (Supplementary Figure S2). Bone mineral density (BMD) measurements of the femoral neck (FN), total hip (TH), and lumbar (L) 1–4, including absolute values, *Z*-scores, and *T*-scores, were obtained using GE Healthcare dual-energy X-ray absorptiometry (DXA) scanners. Although scanner models differed across centers, all measurements were conducted by trained technicians according to standardized procedures. Routine quality control procedures were performed according to the manufacturer’s recommendations, and cross-calibration within the same reference database was used to reduce inter-device measurement variability.

### 2.3 Diagnostic criteria

Radiographic OA was defined as a Kellgren–Lawrence (K-L) grade ≥2 (12). Site-specific OA was defined as a K-L grade ≥2 in either side of the assessed joint, including the knee, hip, or hand. Overall OA was defined as the presence of radiographic OA in any of the assessed sites. All radiographs were independently graded by three experienced orthopedic surgeons who were blinded to participants’ information. Disagreements in K-L grades were resolved through discussion and consensus. OP diagnosis was based on the following criteria (any one of the three sufficed for diagnosis): (1) fragility fractures of the hip or vertebral body; (2) T-score ≤ −2.5 at the FN, TH, or L1–4; (3) −2.5 < T-score < −1.0 at the FN, TH, or L1–4, accompanied by fragility fractures of the proximal humerus, pelvis, or distal forearm. OPF diagnosis followed previously established criteria (11).

### 2.4 Statistical analysis

Standardized rates were calculated using data from the seventh national census of China, with 95% confidence intervals (CIs) estimated via the normal approximation method. Continuous variables were analyzed using the Mann–Whitney *U* test or Kruskal–Wallis test, while categorical variables were analyzed using the chi-square (trend) test or Fisher’s exact test. Statistical significance was set at *p* < 0.05. Multivariable logistic regression was conducted using a stepwise method to compute odds ratios (ORs), with a significant level of 0.1 in the regression model. Prevalence estimates and regression analyses were based on the final analytical sample, whereas sampling weights and variance estimation accounting for clustering were not incorporated. Data analysis was performed using SPSS 25.0 (SPSS Inc., IL, USA).

## 3 Results

### 3.1 Demographic characteristics

**Figure 1:**
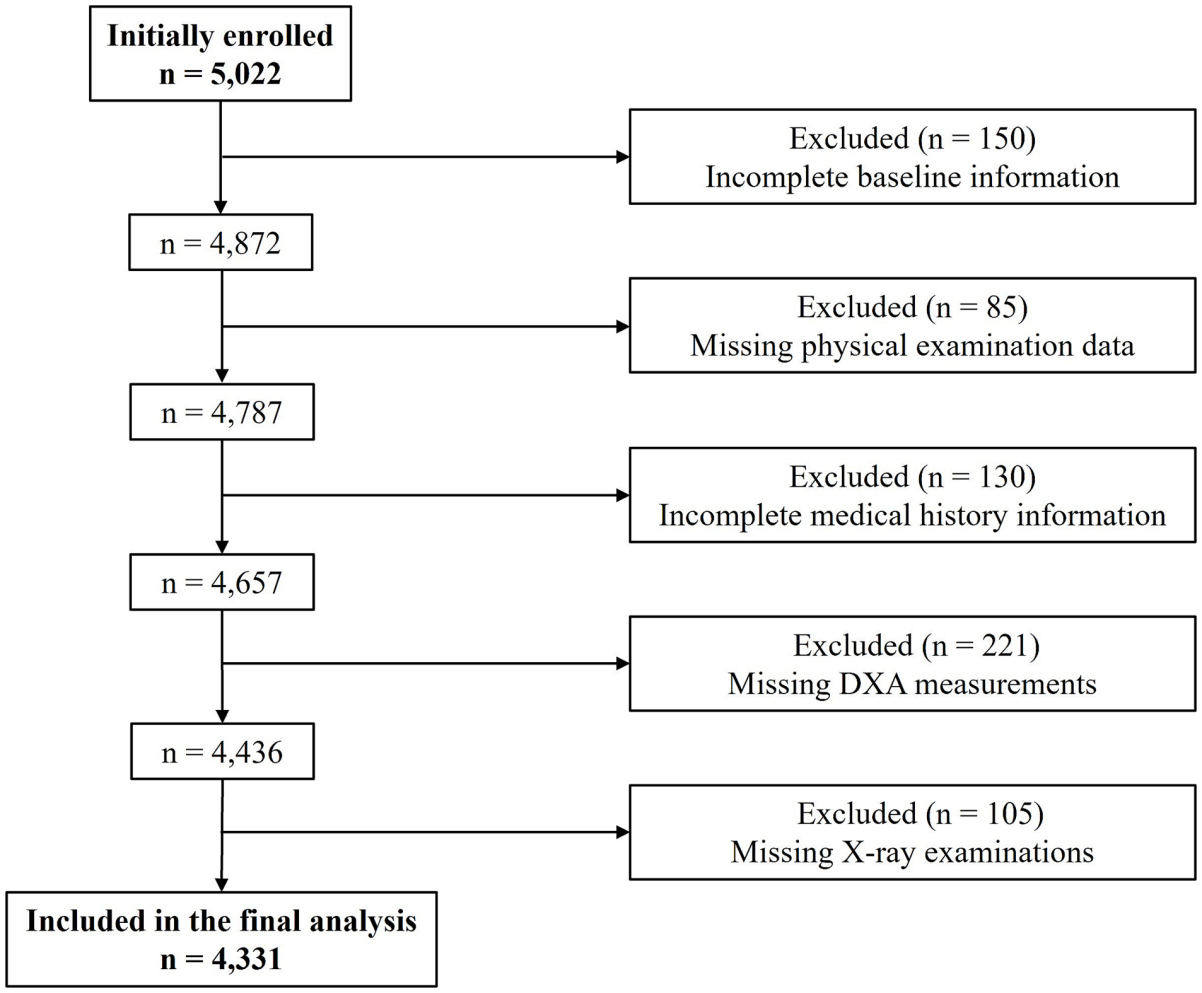
Flow diagram of participant selection. A total of 5,022 individuals aged 60 years or older were initially enrolled. After sequential exclusion, 4,331 participants were included in the final analysis. DXA, dual-energy X-ray absorptiometry.

A total of 5,022 individuals aged 60 years and older were initially enrolled in this study. Sequential exclusions were performed for participants with incomplete baseline information (*n* = 150), missing physical examination data (*n* = 85), incomplete medical history information (*n* = 130), missing DXA measurements (*n* = 221), and missing X-ray examinations (*n* = 105). Finally, 4,331 participants with complete data were included in the final analyses (Figure 1). The average age of the participants was 69.86 ± 6.04 years. Among them, 54.7% were aged 60–69 years (*n* = 2,368), 38.0% were aged 70–79 years (*n* = 1,644), and 7.4% were aged 80 years or older (*n* = 319) (Supplementary Table S1).

### 3.2 Prevalence of OA

**Figure 2:**
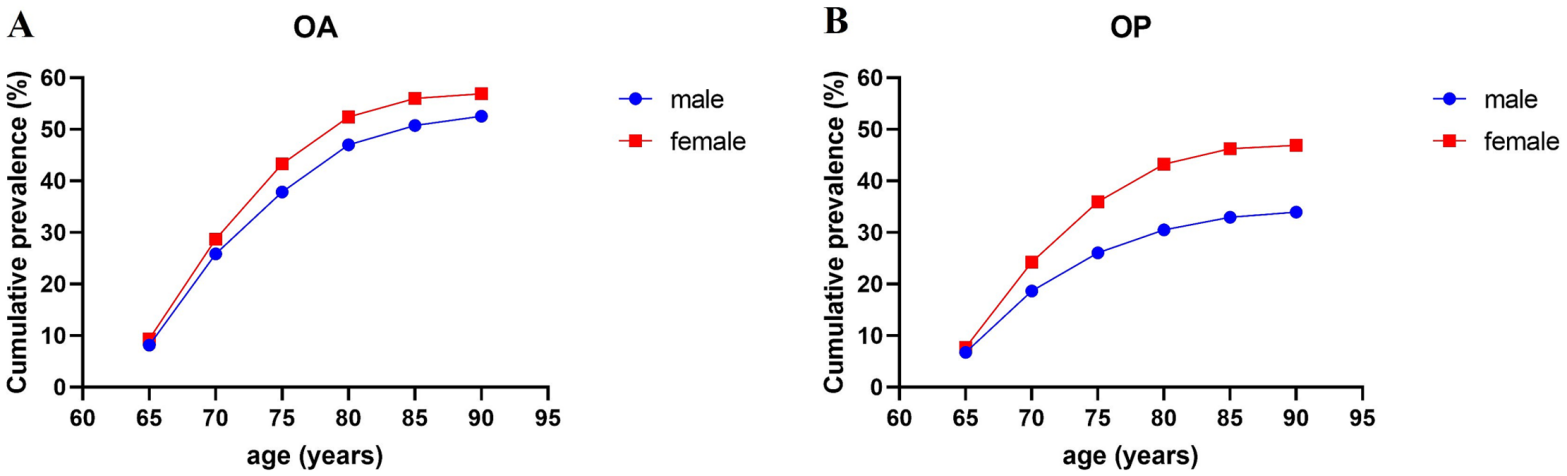
Age-specific cumulative prevalence of OA and OP in older Chinese adults. **(A)** Cumulative prevalence of OA in men and women. **(B)** Cumulative prevalence of OP in men and women. The prevalence of both OA and OP increased progressively with age and was consistently higher in women than in men across all age groups. OA, osteoarthritis; OP, osteoporosis.

Radiographic evaluations using K-L grading were performed on the knees, hips, and hands. There were significant differences in knee K-L grades between males and females (left knee: *p* < 0.001; right knee: *p* = 0.006) (Supplementary Table S2). K-L grades differed significantly across age groups for all joints (*p* < 0.001) (Supplementary Table S3). The overall prevalence of OA was 55.7% (95% CI, 54.2–57.1%), with a significantly higher prevalence in females (57.2%; 95% CI, 55.3–59.0%) compared to males (52.9%; 95% CI, 50.4–55.4%) (*p* = 0.007) (Figure 2A). OA prevalence increased with age (*p* < 0.001), with prevalence of 50.7% (95% CI, 48.7–52.7%) in the 60–69 age group, 60.2% (95% CI, 57.8–62.5%) in the 70–79 group, and 69.6% (95% CI, 64.5–74.6%) in those aged 80 and above (Table 1). The prevalence of OA in different joints increased significantly with age (knee and hand: *p* < 0.001; hip: *p* = 0.017) (Table 2). OA was most prevalent in Southwest China (knee), Northwest China (hip) and East China (hand), and least in East China (knee) and South China (hip, hand) (*p* < 0.001) (Table 2).

**Table 1:** Prevalence of OA and OP in older Chinese adults with different sexes and ages.

Diagnosis	Age (years)	Prevalence, % (95%*CI*)	
		Male	Female
OA	60–69	49.5 (46.0–53.0)	51.3 (48.8–53.8)
	70–79	55.2 (51.2–59.2)	62.9 (60.0–65.8)
	≥80	62.7 (54.7–70.7)	75.1 (68.7–81.5)
	Total	52.9 (50.4–55.4)	57.2 (55.4–59.0)
	*p* ^a^	<0.001	<0.001
OP	60–69	35.7 (32.4–39.0)	43.3 (40.9–45.7)
	70–79	30.9 (27.1–34.7)	50.4 (47.4–53.4)
	≥80	38.0 (30.0–46.0)	61.0 (53.8–68.2)
	Total	34.1 (31.7–36.5)	47.1 (45.3–48.9)
	*p* ^a^	0.546	<0.001

a Chi-square trend test. OA, osteoarthritis; OP, osteoporosis; CI, confidence interval.

**Table 2:** Prevalence of knee, hip, and hand OA in older Chinese adults with different ages or regions.

Variables	Groups	Prevalence, % (95%*CI*)		
		Knee OA	Hip OA	Hand OA
Age (years)	60–69	33.4 (31.5–35.3)	24.5 (22.8–26.2)	26.1 (24.3–27.9)
	70–79	38.9 (36.5–41.3)	27.0 (24.9–29.1)	34.0 (31.7–36.3)
	≥80	47.3 (41.8–52.8)	29.5 (24.5–34.5)	41.7 (36.3–47.1)
	Total	36.5 (35.1–37.9)	25.8 (24.5–27.1)	30.3 (28.9–31.7)
	*p* ^a^	<0.001	0.017	<0.001
Regions	Southwest	44.9 (41.9–48.0)	32.5 (29.6–35.4)	26.9 (24.2–29.6)
	North	42.0 (39.1–44.9)	21.5 (19.1–23.9)	34.7 (31.9–37.5)
	East	17.7 (14.7–20.7)	21.3 (18.1–24.5)	46.6 (42.7–50.5)
	Northwest	37.5 (33.2–41.8)	44.9 (40.5–49.3)	26.6 (22.7–30.5)
	South	33.1 (30.2–36.0)	17.7 (15.4–20.0)	20.5 (18.0–23.0)
	Total	36.5 (35.1–37.9)	25.8 (24.5–27.1)	30.3 (28.9–31.7)
	*p* ^b^	<0.001	<0.001	<0.001

a Chi-square trend test.

b Chi-square test. OA, osteoarthritis; CI, confidence interval.

### 3.3 Prevalence of OP

BMD values across the FN, TH, and L1–4 stratified by sexes and ages are presented in Supplementary Table S4. Based on BMD values and/or history of fragility fractures, the overall prevalence of OP among individuals aged 60 and above was 42.6% (95% CI, 41.1–44.1%). OP prevalence was significantly higher in females (47.1%; 95% CI, 45.3–48.9%) than in males (34.1%; 95% CI, 31.7–36.5%) (*p* < 0.001) (Figure 2B). OP prevalence increased significantly with age (*p* = 0.001): 40.8% (95% CI, 38.8–42.8%) in those aged 60–69, 43.5% (95% CI, 41.1–45.9%) in those aged 70–79, and 50.8% (95% CI, 45.3–56.3%) in those aged 80 and above (Table 1). Region-specific standardized rates indicated the highest OP prevalence in North China and the lowest in East China (*p* < 0.001) (Supplementary Table S5).

### 3.4 Factors associated with OA and OP

Multivariable logistic regression analysis identified several factors associated with OA and OP. Female sex, increasing age, manual occupation, and coexisting OP were associated with higher odds of overall OA, while regular vitamin D supplementation was associated with lower odds of OA (Table 3). In site-specific analyses, higher BMI, manual occupation, and greater FTA were associated with higher odds of knee OA (Supplementary Table S6). For hip OA, higher BMI was associated with lower odds, whereas manual occupation, exercise frequency, CEA, and FTA were associated with higher odds (Supplementary Table S7). For hand OA, multiple types of exercise were associated with lower odds (Supplementary Table S8).

**Table 3:** Multivariable logistic regression analysis of OA in older Chinese adults.

Variables	Groups	*p*	*OR* (95%*CI*)
Sex	Female	0.017	1.17 (1.03–1.34)
Age (years)	60–69	NA	1 [Reference]
	70–79	<0.001	1.41 (1.23–1.60)
	≥80	<0.001	2.28 (1.76–2.95)
BMI (kg/m^2^)	18.5–24	NA	1 [Reference]
	<18.5	0.078	0.70 (0.47–1.04)
	≥24	0.327	1.07 (0.94–1.21)
Regions	Southwest	NA	1 [Reference]
	North	0.056	1.20 (1.00–1.44)
	East	0.106	0.84 (0.68–1.04)
	Northwest	0.658	1.05 (0.84–1.32)
	South	0.066	0.83 (0.68–1.01)
Occupation	Manual	0.015	1.18 (1.03–1.34)
Vitamin D	Yes	0.023	0.81 (0.68–0.97)
Activated vitamin D	Yes	0.096	0.84 (0.68–1.03)
HL	Yes	0.070	1.15 (0.99–1.33)
IBD	Yes	0.072	1.39 (0.97–1.98)
OP	Yes	0.005	1.20 (1.06–1.36)

OA, osteoarthritis; OR, odds ratio; CI, confidence interval; BMI, body mass index; HL, hyperlipidemia; IBD, inflammatory bowel disease; OP, osteoporosis.

Factors associated with higher odds of OP included female sex, increasing age, height loss, history of traumatic spinal fracture, and coexisting OA. Higher BMI, higher education level, and regular vitamin D supplementation were associated with lower odds of OP (Table 4). Earlier age of menopause was associated with higher odds of OP among women, while CHF was associated with higher odds of OP among men (Supplementary Tables S9, S10).

**Table 4:** Multivariable logistic regression analysis of OP in older Chinese adults.

Variables	Groups	*p*	*OR* (95%*CI*)
Sex	Female	<0.001	1.60 (1.40–1.83)
Age (years)	60–69	NA	1 [Reference]
	70–79	0.078	1.13 (0.99–1.30)
	≥80	<0.001	1.58 (1.23–2.03)
BMI (kg/m^2^)	18.5–24	NA	1 [Reference]
	<18.5	0.180	1.31 (0.88–1.95)
	≥24	<0.001	0.75 (0.66–0.85)
Height loss (cm)	NA	0.001	1.06 (1.02–1.09)
Regions	Southwest	NA	1 [Reference]
	North	<0.001	1.75 (1.45–2.11)
	East	<0.001	0.55 (0.44–0.69)
	Northwest	0.595	1.06 (0.85–1.34)
	South	0.146	0.86 (0.70–1.05)
Education	Primary	NA	1 [Reference]
	Secondary	0.366	0.93 (0.79–1.09)
	Senior	0.001	0.69 (0.56–0.86)
Vitamin D	Yes	0.002	0.81 (0.70–0.92)
History of traumatic spinal fracture	Yes	<0.001	4.45 (2.53–7.84)
OA	Yes	0.007	1.19 (1.05–1.35)

OP, osteoporosis; OR, odds ratio; CI, confidence interval; BMI, body mass index; OA, osteoarthritis.

## 4 Discussion

In this large-scale, multicenter community-based cross-sectional study of adults aged 60 years or older from five Chinese cities, we observed a high prevalence of both OA and OP. The prevalence of OA and OP was 55.7 and 42.6%, respectively. Notably, our study suggested a coexistence between OA and OP in older adults. We also identified geographic variations in the prevalence, as well as potential associated factors. To our knowledge, this is one of the few multicenter epidemiological studies in China to simultaneously investigate OA and OP in an older population.

In our study, the prevalence of OA and OP appeared higher than those reported in previous studies; however, these comparisons should be interpreted cautiously because of differences in study populations, age ranges, diagnostic criteria, anatomical sites, and assessment methods. For example, the Framingham OA Study in the US reported a prevalence of hip OA of 19.6% among individuals aged 50 years and older (13). Data from the Osteoarthritis Initiative (OAI) suggested a hand OA prevalence of 41.4% (14). In addition, a systematic review and meta-analysis reported a global OP prevalence of 19.7% (15). Several factors may partly explain the differences between our findings and previous reports: (1) Our study was a multicenter cross-sectional survey focusing on adults aged 60 years or older in China, and differences in ethnicity, socioeconomic status, lifestyle, and other population characteristics may have influenced the results; (2) Our study evaluated OA across commonly affected sites (knee, hip, and hand) rather than a single joint; and (3) OP diagnosis in our study was based on DXA results and/or history of fragility fractures. Relying solely on BMD may lead to underdiagnosis; therefore, thoracolumbar spine X-rays were also performed to identify vertebral fragility fractures (16, 17).

Several studies have reported the prevalence of knee OA and OP is relatively high in Southwest China, which is consistent with our findings (7, 10). This may be partly related to differences in occupational patterns, terrain, lifestyle, socioeconomic status, and healthcare access across regions. Interestingly, our study also revealed a higher prevalence of OP in North China. Reduced sunlight exposure, high salt intake, and sedentary urban lifestyles may contribute to poorer bone health (18, 19). Moreover, hand OA was more common in eastern regions, which may reflect differences in occupational or lifestyle-related hand use. Similar trends have been reported worldwide, with hand OA prevalence in high-income regions such as North America even exceeding that of knee OA (20). Because participants were recruited from five cities, these regional differences should be interpreted cautiously and require confirmation in larger studies.

OA and OP are among the most common degenerative skeletal disorders in the older adults, and our regression analysis showed an association between the two conditions. Although their clinical symptoms and pathophysiological mechanisms differ, they share some demographic factors, such as age and sex (21). The relationship between OA and OP remains controversial. Some previous studies have suggested an inverse association between OA and OP, partly due to subchondral bone changes in OA that may increase measured BMD and the distinct effects of BMI on these two diseases (22, 23). However, recent evidence supports the coexistence of OA and OP. For example, OP-related subchondral bone loss may accelerate OA progression and inflammatory mediators involved in OA may also regulate bone metabolism and aggravate OP (24, 25).

Manual occupation was associated with higher odds of OA. This finding is biologically plausible because long-term occupational loading may contribute to mechanical stress on joints. However, occupation was broadly classified as manual or mental work, which may not fully capture lifetime joint loading, work intensity, or changes in occupation. Vitamin D supplementation was associated with lower odds of both OA and OP in our study. With the increasing prevalence of sedentary lifestyles, vitamin D insufficiency may be common among older adults. Although current clinical evidence does not support vitamin D alone as an effective treatment for OA or OP, regular supplementation may be relevant to bone health in older adults at risk of vitamin D deficiency (26, 27). Previous studies have suggested that vitamin D may be involved in cartilage metabolism and calcium homeostasis, which are relevant to OA and OP.

In older adults, the assessment and management of OA and OP are often complicated by multimorbidity and age-related comorbidities, such as DM, HTN, HL, and COPD (28). In our study, these common comorbidities were not significantly associated with higher odds of OA or OP. For OA, the lack of significant associations with common metabolic comorbidities was consistent with previous studies reported by Dawson and Marshall (29, 30). CHF was associated with higher odds of OP among men. Previous studies have suggested that heart failure is related to impaired bone health, including low BMD, OP, and increased fracture risk (31). Potential mechanisms include reduced mobility, frailty or sarcopenia, malnutrition, vitamin D deficiency, sympathetic activation, renal dysfunction, and secondary hyperparathyroidism (32, 33). The association observed only in men may reflect sex-related differences in the factors associated with OP. However, this finding should be interpreted cautiously because it was based on cross-sectional subgroup analysis.

Several limitations should be acknowledged. First, due to the cross-sectional design, causal relationships between the observed associated factors and OA or OP cannot be established. Therefore, factors associated with higher or lower odds of OA or OP should not be interpreted as confirmed risk or protective factors. Second, the age-related increase in lumbar spine BMD among men should also be interpreted cautiously. Lumbar spine DXA measurements in older adults may be influenced by degenerative spinal changes, osteophytes, and vascular calcification, which can elevate measured BMD. This may partly explain the higher lumbar spine BMD observed in older men. Finally, although prevalence estimates were standardized using census data, sampling weights and design-based variance estimation accounting for clustering were not incorporated into the statistical analyses. Therefore, the precision of prevalence estimates and the CIs of regression results may be affected. Our findings should be interpreted as reflecting this multicenter community-based population rather than nationally representative estimates for all older Chinese adults.

## 5 Conclusion

This study provided updated evidence on the burden and distribution of OA and OP among adults aged 60 years or older from five Chinese cities, highlighting the importance of early screening and prevention strategies for age-related skeletal disorders.

## Data Availability

The raw data supporting the conclusions of this article will be made available by the authors, without undue reservation.

## References

[1] Beard JR Officer A de Carvalho IA Sadana R Pot AM Michel JP . The world report on ageing and health: a policy framework for healthy ageing. Lancet. (2016) 387:2145–54. doi: 10.1016/s0140-6736(15)00516-4, 26520231 PMC4848186

[2] Annual population data of the National Bureau of Statistics. China. Available online at: https://www.stats.gov.cn/sj/pcsj/.

[3] Partridge L Deelen J Slagboom PE. Facing up to the global challenges of ageing. Nature. (2018) 561:45–56. doi: 10.1038/s41586-018-0457-8, 30185958

[4] Ren J Song M Zhang W Cai JP Cao F Cao Z . The aging biomarker consortium represents a new era for aging research in China. Nat Med. (2023) 29:2162–5. doi: 10.1038/s41591-023-02444-y, 37468667

[5] Si L Winzenberg TM Jiang Q Chen M Palmer AJ. Projection of osteoporosis-related fractures and costs in China: 2010-2050. Osteoporos Int. (2015) 26:1929–37. doi: 10.1007/s00198-015-3093-2, 25761729

[6] Liu Q Wang S Lin J Zhang Y. The burden for knee osteoarthritis among Chinese elderly: estimates from a nationally representative study. Osteoarthr Cartil. (2018) 26:1636–42. doi: 10.1016/j.joca.2018.07.019, 30130589

[7] Tang X Wang S Zhan S Niu J Tao K Zhang Y . The prevalence of symptomatic knee osteoarthritis in China: results from the China health and retirement longitudinal study. Arthritis Rheumatol. (2016) 68:648–53. doi: 10.1002/art.39465, 26474054

[8] Wang L Yu W Yin X Cui L Tang S Jiang N . Prevalence of osteoporosis and fracture in China: the China osteoporosis prevalence study. JAMA Netw Open. (2021) 4:e2121106. doi: 10.1001/jamanetworkopen.2021.21106, 34398202 PMC8369359

[9] Zeng Q Li N Wang Q Feng J Sun D Zhang Q . The prevalence of osteoporosis in China, a Nationwide, multicenter DXA survey. J Bone Miner Res. (2019) 34:1789–97. doi: 10.1002/jbmr.3757, 31067339

[10] Cheng X Zhao K Zha X Du X Li Y Chen S . Opportunistic screening using low-dose CT and the prevalence of osteoporosis in China: a nationwide, multicenter study. J Bone Miner Res. (2021) 36:427–35. doi: 10.1002/jbmr.4187, 33145809 PMC7988599

[11] Li Q Yang Z Zhu M Li J Lu C Li Z . Prevalence and risk factors of osteoporotic fracture among the elderly population in China: a multicenter cross-sectional study. Int Orthop. (2024) 48:1323–30. doi: 10.1007/s00264-024-06145-0, 38467869

[12] Schiphof D Boers M Bierma-Zeinstra SM. Differences in descriptions of Kellgren and Lawrence grades of knee osteoarthritis. Ann Rheum Dis. (2008) 67:1034–6. doi: 10.1136/ard.2007.079020 18198197

[13] Kim C Linsenmeyer KD Vlad SC Guermazi A Clancy MM Niu J . Prevalence of radiographic and symptomatic hip osteoarthritis in an urban United States community: the Framingham osteoarthritis study. Arthritis Rheumatol. (2014) 66:3013–7. doi: 10.1002/art.38795, 25103598 PMC4211973

[14] Eaton CB Schaefer LF Duryea J Driban JB Lo GH Roberts MB . Prevalence, incidence, and progression of radiographic and symptomatic hand osteoarthritis: the osteoarthritis initiative. Arthritis Rheumatol. (2022) 74:992–1000. doi: 10.1002/art.42076, 35077023

[15] Xiao PL Cui AY Hsu CJ Peng R Jiang N Xu XH . Global, regional prevalence, and risk factors of osteoporosis according to the World Health Organization diagnostic criteria: a systematic review and meta-analysis. Osteoporos Int. (2022) 33:2137–53. doi: 10.1007/s00198-022-06454-3, 35687123

[16] Nuti R Brandi ML Checchia G Di Munno O Dominguez L Falaschi P . Guidelines for the management of osteoporosis and fragility fractures. Intern Emerg Med. (2019) 14:85–102. doi: 10.1007/s11739-018-1874-2, 29948835 PMC6329834

[17] Siris ES Adler R Bilezikian J Bolognese M Dawson-Hughes B Favus MJ . The clinical diagnosis of osteoporosis: a position statement from the National Bone Health Alliance Working Group. Osteoporos Int. (2014) 25:1439–43. doi: 10.1007/s00198-014-2655-z, 24577348 PMC3988515

[18] Wang Y Tao Y Hyman ME Li J Chen Y. Osteoporosis in China. Osteoporos Int. (2009) 20:1651–62. doi: 10.1007/s00198-009-0925-y, 19415374

[19] Yu F Xia W. The epidemiology of osteoporosis, associated fragility fractures, and management gap in China. Arch Osteoporos. (2019) 14:32. doi: 10.1007/s11657-018-0549-y, 30848398

[20] Long H Liu Q Yin H Wang K Diao N Zhang Y . Prevalence trends of site-specific osteoarthritis from 1990 to 2019: findings from the global burden of disease study 2019. Arthritis Rheumatol. (2022) 74:1172–83. doi: 10.1002/art.42089, 35233975 PMC9543105

[21] Huang K Cai H. The interplay between osteoarthritis and osteoporosis: mechanisms, implications, and treatment considerations - a narrative review. Exp Gerontol. (2024) 197:112614. doi: 10.1016/j.exger.2024.112614, 39442896

[22] Radin EL Rose RM. Role of subchondral bone in the initiation and progression of cartilage damage. Clin Orthop Relat Res. (1986) 213:34–40. doi: 10.1097/00003086-198612000-00005, 3780104

[23] Oliveira MC Vullings J van de Loo FAJ. Osteoporosis and osteoarthritis are two sides of the same coin paid for obesity. Nutrition. (2020) 70:110486. doi: 10.1016/j.nut.2019.04.001, 31655472

[24] Hunter DJ Bierma-Zeinstra S. Osteoarthritis. Lancet. (2019) 393:1745–59. doi: 10.1016/s0140-6736(19)30417-9 31034380

[25] Bultink IE Lems WF. Osteoarthritis and osteoporosis: what is the overlap? Curr Rheumatol Rep. (2013) 15:328. doi: 10.1007/s11926-013-0328-0, 23508809

[26] Kuswanto W Baker MC. Repurposing drugs for the treatment of osteoarthritis. Osteoarthr Cartil. (2024) 32:886–95. doi: 10.1016/j.joca.2024.05.008, 38821468

[27] Chevalley T Brandi ML Cashman KD Cavalier E Harvey NC Maggi S . Role of vitamin D supplementation in the management of musculoskeletal diseases: update from an European Society of Clinical and Economical Aspects of osteoporosis, osteoarthritis and musculoskeletal diseases (ESCEO) working group. Aging Clin Exp Res. (2022) 34:2603–23. doi: 10.1007/s40520-022-02279-6, 36287325 PMC9607746

[28] Fuggle N Laslop A Rizzoli R Al-Daghri N Alokail M Balkowiec-Iskra E . Treatment of osteoporosis and osteoarthritis in the oldest old. Drugs. (2025) 85:343–60. doi: 10.1007/s40265-024-02138-w, 39969778 PMC11891106

[29] Dawson LP Fairley JL Papandony MC Hussain SM Cicuttini FM Wluka AE. Is abnormal glucose tolerance or diabetes a risk factor for knee, hip, or hand osteoarthritis? A systematic review. Semin Arthritis Rheum. (2018) 48:176–89. doi: 10.1016/j.semarthrit.2018.02.008 29550110

[30] Marshall M Peat G Nicholls E Myers HL Mamas MA van der Windt DA. Metabolic risk factors and the incidence and progression of radiographic hand osteoarthritis: a population-based cohort study. Scand J Rheumatol. (2019) 48:52–63. doi: 10.1080/03009742.2018.1459831, 29952684 PMC6319183

[31] Aluoch AO Jessee R Habal H Garcia-Rosell M Shah R Reed G . Heart failure as a risk factor for osteoporosis and fractures. Curr Osteoporos Rep. (2012) 10:258–69. doi: 10.1007/s11914-012-0115-2, 22915207

[32] Spoladore R Ciampi CM Ossola P Sultana A Spreafico LP Farina A . Heart failure and osteoporosis: shared challenges in the aging population. J Cardiovasc Dev Dis. (2025) 12:69. doi: 10.3390/jcdd12020069, 39997503 PMC11856909

[33] Guan Z Yuan W Jia J Zhang C Zhu J Huang J . Bone mass loss in chronic heart failure is associated with sympathetic nerve activation. Bone. (2023) 166:116596. doi: 10.1016/j.bone.2022.116596, 36307018

