# Supplementary material for: Prevalence and associated factors of osteoarthritis and osteoporosis among Chinese adults aged 60 years or older: a multicenter cross-sectional study
Source: Front Public Health. 2026 Jul 30;14:1905264. doi: 10.3389/fpubh.2026.1905264 (PMC13467993; doi:10.3389/fpubh.2026.1905264)
Supplement: Supplementary file 1 [file Data_Sheet_1.PDF]

Supplementary materials

Figure S1 Angle measurement of hip joint 2

Figure S2 Angle measurement of knee joint........ 2

Table S1 Demographic characteristics of the participants 3

Table S2 Kellgren and Lawrence grades of older Chinese adults by sex 4

Table S3 Kellgren and Lawrence grades of older Chinese adults by age 5

Table S4 Mean BMD in older Chinese adults with different sexes and ages 6

Table S5 Prevalence of OP in older Chinese adults with different regions 7

Table S6 Multivariable logistic regression analysis of knee OA in older Chinese adults........ 8

Table S7 Multivariable logistic regression analysis of hip OA in older Chinese adults........ 9

Table S8 Multivariable logistic regression analysis of hand OA in older Chinese adults 10

Table S9 Multivariable logistic regression analysis of OP in older Chinese women 11

Table S10 Multivariable logistic regression analysis of OP in older Chinese men 12

Figure S1 Angle measurement of hip joint

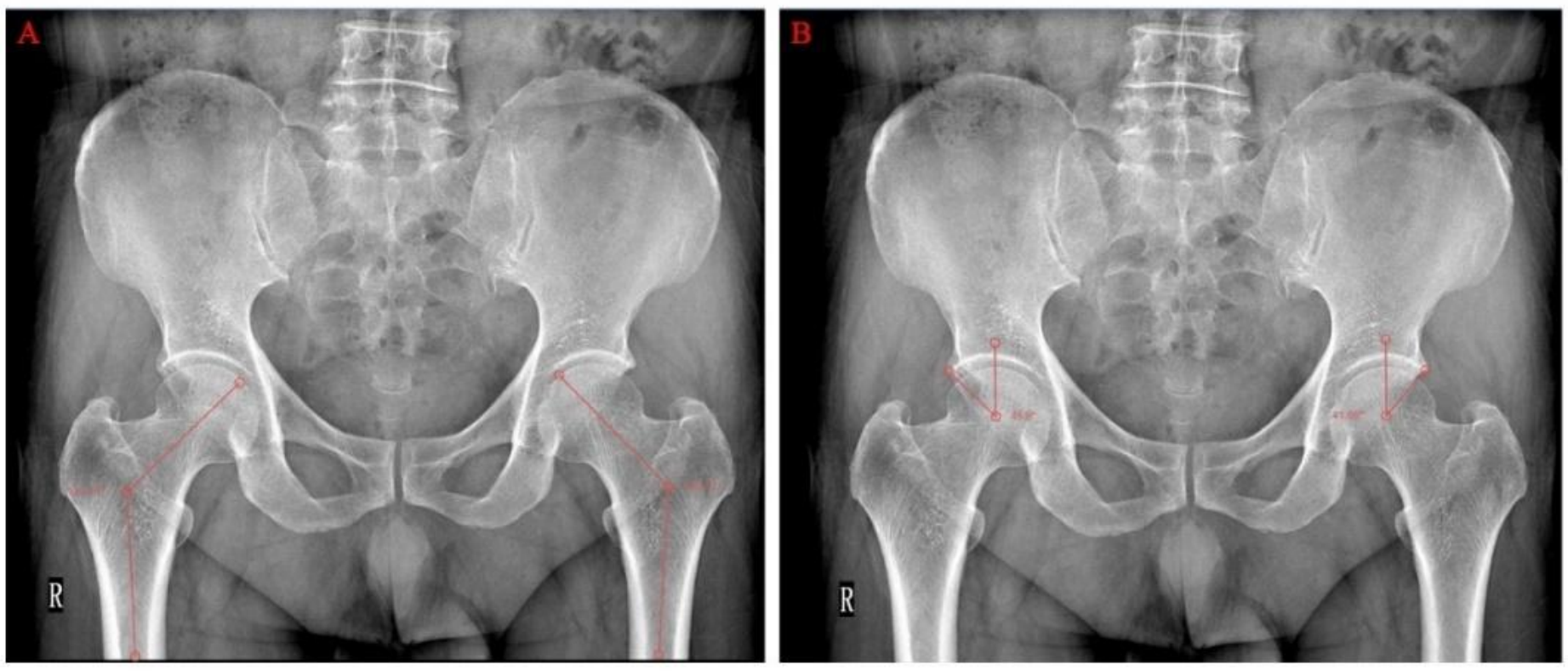

(A) Collo-diaphyseal angle (CDA); (B) Center-edge angle (CEA).

Figure S2 Angle measurement of knee joint

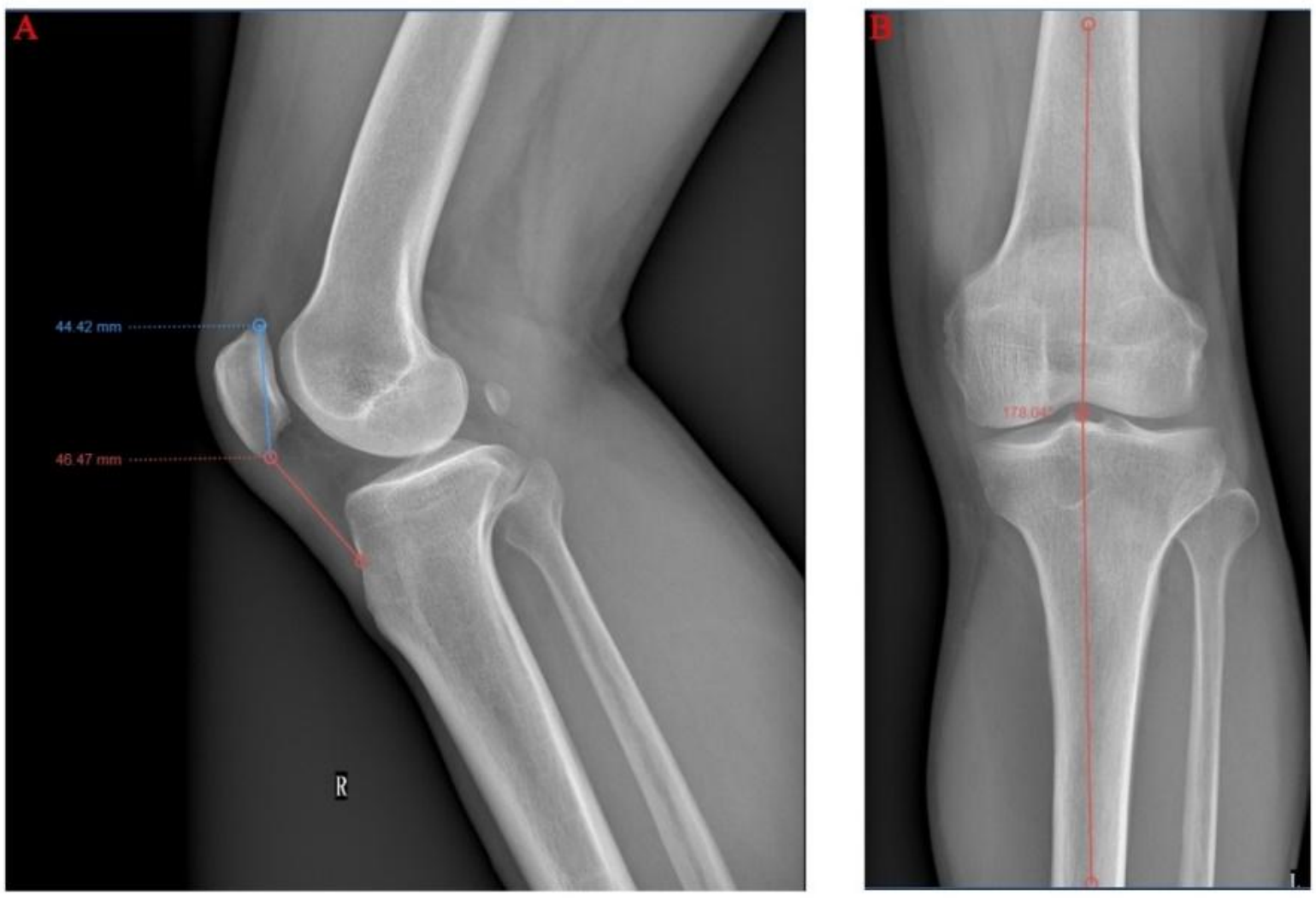

(A) Insall-Salvati index; (B) Femoral tibial angle (FTA).

Table S1 Demographic characteristics of the participants

Variables	Groups	Total, n	Male, n	Female, n
**Age (years)**	60–69	2368	792	1576
	70–79	1644	582	1062
	≥80	319	142	177
**BMI (kg/m^2)**	18.5–24	2036	675	1361
	<18.5	111	36	75
	≥24	2184	805	1379
**Regions**	Southwest	1023	345	678
	North	1151	316	835
	East	633	218	415
	Northwest	488	206	282
	South	1036	431	605
**Occupation**	Mental	2515	815	1700
	Manual	1816	701	1115

BMI, body mass index.

Table S2 Kellgren and Lawrence grades of older Chinese adults by sex

Sites	Sex	Grade 0, n	Grade 1, n	Grade 2, n	Grade 3, n	Grade 4, n	Total, n	*P*[a]
**Left hand**	Male	642	518	269	71	16	1516	0.032
	Female	1190	864	559	151	51	2815	
	Total	1832	1382	828	222	67	4331	
**Right hand**	Male	631	510	283	77	15	1516	0.066
	Female	1178	860	567	160	50	2815	
	Total	1809	1370	850	237	65	4331	
**Left knee**	Male	540	546	302	107	21	1516	<0.001
	Female	907	929	690	226	63	2815	
	Total	1447	1475	992	333	84	4331	
**Right knee**	Male	538	530	299	126	23	1516	0.006
	Female	914	918	658	259	66	2815	
	Total	1452	1448	957	385	89	4331	
**Left hip**	Male	673	522	243	71	7	1516	0.324
	Female	1267	938	449	131	30	2815	
	Total	1940	1460	692	202	37	4331	
**Right hip**	Male	669	487	273	71	16	1516	0.507
	Female	1308	899	456	124	28	2815	
	Total	1977	1386	729	195	44	4331	

[a]Chi-square test.

Table S3 Kellgren and Lawrence grades of older Chinese adults by age

Sites	Age (years)	Grade 0, n	Grade 1, n	Grade 2, n	Grade 3, n	Grade 4, n	Total, n	P^a
**Left hand**	60–69	1122	724	392	99	31	2368	<0.001
	70–79	599	564	353	100	28	1644	
	≥80	111	94	83	23	8	319	
	Total	1832	1382	828	222	67	4331	
**Right hand**	60–69	1111	712	414	100	31	2368	<0.001
	70–79	591	570	349	110	24	1644	
	≥80	107	88	87	27	10	319	
	Total	1809	1370	850	237	65	4331	
**Left knee**	60–69	908	761	471	180	48	2368	<0.001
	70–79	451	620	427	121	25	1644	
	≥80	88	94	94	32	11	319	
	Total	1447	1475	992	333	84	4331	
**Right knee**	60–69	907	737	477	195	52	2368	<0.001
	70–79	457	614	394	151	28	1644	
	≥80	88	97	86	39	9	319	
	Total	1452	1448	957	385	89	4331	
**Left hip**	60–69	1125	750	332	139	22	2368	<0.001
	70–79	666	618	297	52	11	1644	
	≥80	149	92	63	11	4	319	
	Total	1940	1460	692	202	37	4331	
**Right hip**	60–69	1151	710	351	132	24	2368	<0.001
	70–79	680	589	312	49	14	1644	
	≥80	146	87	66	14	6	319	
	Total	1977	1386	729	195	44	4331	

[a]Chi-square test.

Table S4 Mean BMD in older Chinese adults with different sexes and ages

Sites	Age (years)	Male		Female	
		n	BMD, g/cm^2 (95%*CI*)	n	BMD, g/cm^2 (95%*CI*)
**FN**	60–69	792	0.865 (0.853–0.878)	1576	0.788 (0.782–0.795)
	70–79	582	0.875 (0.861–0.888)	1062	0.755 (0.747–0.763)
	≥80	142	0.863 (0.837–0.890)	177	0.712 (0.690–0.735)
	Total	1516	0.868 (0.860–0.877)	2815	0.771 (0.766–0.776)
**TH**	60–69	792	0.962 (0.948–0.976)	1576	0.873 (0.866–0.880)
	70–79	582	0.969 (0.953–0.984)	1062	0.835 (0.826–0.844)
	≥80	142	0.968 (0.941–0.996)	177	0.801 (0.780–0.822)
	Total	1516	0.965 (0.955–0.975)	2815	0.854 (0.849–0.860)
**L1–4**	60–69	792	1.071 (1.055–1.087)	1576	0.970 (0.961–0.979)
	70–79	582	1.114 (1.096–1.132)	1062	0.946 (0.935–0.958)
	≥80	142	1.205 (1.163–1.248)	177	0.951 (0.923–0.979)
	Total	1516	1.100 (1.088–1.112)	2815	0.960 (0.953–0.967)

BMD, bone mineral density; FN, femoral neck; TH, total hip; L, lumbar; *CI*, confidence interval.

Table S5 Prevalence of OP in older Chinese adults with different regions

Regions	Prevalence, % (95%*CI*)
**Southwest**	43.4 (40.4–46.4)
**North**	52.4 (49.5–55.3)
**East**	30.8 (27.2–34.4)
**Northwest**	43.0 (38.6–47.4)
**South**	37.7 (34.7–40.7)
**Total**	42.6 (41.1–44.1)
***P*[a]**	<0.001

[a]Chi-square test. OP, osteoporosis; *CI*, confidence interval.

Table S6 Multivariable logistic regression analysis of knee OA in older Chinese adults

Variables	Groups	*P*	*OR* (95%*CI*)
**Sex**	Female	<0.001	1.31 (1.14–1.51)
**Age (years)**	60–69	NA	1 [Reference]
	70–79	<0.001	1.30 (1.13–1.50)
	≥80	<0.001	1.89 (1.47–2.42)
**BMI (kg/m^2)**	18.5–24	NA	1 [Reference]
	<18.5	0.358	0.81 (0.52–1.26)
	≥24	0.033	1.16 (1.01–1.32)
**Regions**	Southwest	NA	1 [Reference]
	North	0.622	0.96 (0.80–1.14)
	East	<0.001	0.19 (0.15–0.25)
	Northwest	0.015	0.75 (0.59–0.95)
	South	<0.001	0.62 (0.51–0.75)
**Occupation**	Manual	0.002	1.25 (1.09–1.43)
**Vitamin D**	Yes	0.016	0.84 (0.73–0.97)
**FTA**	NA	<0.001	1.08 (1.05–1.10)

OA, osteoarthritis; *OR*, odds ratio; *CI*, confidence interval; BMI, body mass index; FTA, femoral tibial angle.

Table S7 Multivariable logistic regression analysis of hip OA in older Chinese adults

Variables	Groups	*P*	*OR* (95%*CI*)
**Age (years)**	60–69	NA	1 [Reference]
	70–79	0.816	0.98 (0.84–1.15)
	≥80	0.007	1.47 (1.11–1.95)
**BMI (kg/m^2)**	18.5–24	NA	1 [Reference]
	<18.5	0.686	1.10 (0.70–1.74)
	≥24	0.017	0.83 (0.71–0.97)
**Regions**	Southwest	NA	1 [Reference]
	North	<0.001	0.56 (0.45–0.69)
	East	<0.001	0.50 (0.39–0.65)
	Northwest	<0.001	2.16 (1.70–2.74)
	South	<0.001	0.55 (0.43–0.70)
**Occupation**	Manual	0.038	1.18 (1.01–1.39)
**Exercise type**	Multiple	0.053	0.84 (0.70–1.00)
**Exercise frequency**	NA	0.004	1.05 (1.01–1.08)
**CEA**	NA	<0.001	1.03 (1.02–1.04)
**FTA**	NA	<0.001	1.16 (1.13–1.20)

OA, osteoarthritis; *OR*, odds ratio; *CI*, confidence interval; BMI, body mass index; CEA, Center-edge angle; FTA, femoral tibial angle.

Table S8 Multivariable logistic regression analysis of hand OA in older Chinese adults

Variables	Groups	*P*	*OR* (95%*CI*)
**Age (years)**	60–69	NA	1 [Reference]
	70–79	<0.001	1.31 (1.14–1.52)
	≥80	<0.001	2.06 (1.61–2.64)
**BMI (kg/m^2)**	18.5–24	NA	1 [Reference]
	<18.5	0.150	0.71 (0.45–1.13)
	≥24	0.133	1.11 (0.97–1.27)
**Regions**	Southwest	NA	1 [Reference]
	North	<0.001	1.55 (1.28–1.87)
	East	<0.001	2.21 (1.78–2.75)
	Northwest	0.785	0.97 (0.75–1.24)
	South	0.042	0.80 (0.65–0.99)
**Vitamin D**	Yes	<0.001	0.75 (0.64–0.87)
**Exercise type**	Multiple	0.002	0.77 (0.66–0.91)
**IBD**	Yes	0.078	1.38 (0.97–1.97)
**OP**	Yes	0.017	1.18 (1.03–1.36)

OA, osteoarthritis; *OR*, odds ratio; *CI*, confidence interval; BMI, body mass index; IBD, inflammatory bowel disease; OP, osteoporosis.

Table S9 Multivariable logistic regression analysis of OP in older Chinese women

Variables	Groups	*P*	*OR* (95%*CI*)
**Age (years)**	60–69	NA	1 [Reference]
	70–79	0.001	1.34 (1.13–1.58)
	≥80	<0.001	2.04 (1.46–2.85)
**Menopausal age (years)**	NA	0.001	0.97 (0.95–0.99)
**BMI (kg/m²)**	18.5–24	NA	1 [Reference]
	<18.5	0.423	1.22 (0.75–1.96)
	≥24	<0.001	0.66 (0.56–0.77)
**Height loss (cm)**	NA	0.032	1.05 (1.00–1.09)
**Regions**	Southwest	NA	1 [Reference]
	North	0.010	1.34 (1.07–1.67)
	East	<0.001	0.53 (0.40–0.69)
	Northwest	0.002	0.63 (0.47–0.84)
	South	0.019	0.75 (0.58–0.95)
**Vitamin D**	Yes	<0.001	0.72 (0.61–0.86)
**RA**	Yes	0.056	1.39 (0.99–1.96)
**History of traumatic limb fracture**	Yes	0.068	1.28 (0.98–1.67)
**History of traumatic spinal fracture**	Yes	<0.001	3.49 (1.77–6.85)
**OA**	Yes	0.083	1.15 (0.98–1.35)

OP, osteoporosis; *OR*, odds ratio; *CI*, confidence interval; BMI, body mass index; RA, rheumatoid arthritis; OA, osteoarthritis.

Table S10 Multivariable logistic regression analysis of OP in older Chinese men

Variables	Groups	*P*	*OR* (95%*CI*)
**Height loss (cm)**	NA	0.006	1.09 (1.03–1.16)
**Regions**	Southwest	NA	1 [Reference]
	North	<0.001	3.44 (2.40–4.92)
	East	0.018	0.57 (0.36–0.91)
	Northwest	<0.001	2.42 (1.65–3.56)
	South	0.083	1.39 (0.96–2.02)
**Education**	Primary	NA	1 [Reference]
	Secondary	0.024	0.71 (0.53–0.96)
	Senior	<0.001	0.47 (0.32–0.69)
**Exercise type**	Multiple	0.069	1.28 (0.98–1.66)
**CHF**	Yes	0.009	2.16 (1.21–3.83)
**History of traumatic spinal fracture**	Yes	<0.001	7.51 (2.62–21.53)
**OA**	Yes	0.040	1.27 (1.01–1.59)

OP, osteoporosis; *OR*, odds ratio; *CI*, confidence interval; CHF, chronic heart failure; OA, osteoarthritis.
